# Effects of Sub-Chronic MPTP Exposure on Behavioral and Cognitive Performance and the Microbiome of Wild-Type and mGlu8 Knockout Female and Male Mice

**DOI:** 10.3389/fnbeh.2018.00140

**Published:** 2018-07-18

**Authors:** Eileen Ruth S. Torres, Tunde Akinyeke, Keaton Stagaman, Robert M. Duvoisin, Charles K. Meshul, Thomas J. Sharpton, Jacob Raber

**Affiliations:** ^1^Department of Behavioral Neuroscience, Oregon Health & Science University, Portland, OR, United States; ^2^Department of Microbiology, Oregon State University, Corvallis, OR, United States; ^3^Department of Physiology and Pharmacology, Oregon Health & Science University, Portland, OR, United States; ^4^Portland VA Medical Center, Portland, OR, United States; ^5^Department of Statistics, Oregon State University, Corvallis, OR, United States; ^6^Departments of Neurology and Radiation Medicine and Division of Neuroscience, ONPRC, Oregon Health & Science University, Portland, OR, United States

**Keywords:** Parkinson’s disease, metabotropic glutamate receptor, behavioral performance, gut microbiome, tyrosine hydroxylase, beta actin

## Abstract

Motor dysfunction is a hallmark of Parkinson’s disease (PD); however, non-motor symptoms such as gastrointestinal dysfunction often arise prior to motor symptoms. Alterations in the gut microbiome have been proposed as the earliest event in PD pathogenesis. PD symptoms often demonstrate sex differences. Glutamatergic neurotransmission has long been linked to PD pathology. Metabotropic glutamate receptors (mGlu), a family of G protein-coupled receptors, are divided into three groups, with group III mGlu receptors mainly localized presynaptically where they can inhibit glutamate release in the CNS as well as in the gut. Additionally, the gut microbiome can communicate with the CNS via the gut-brain axis. Here, we assessed whether deficiency of metabotropic glutamate receptor 8 (mGlu8), group III mGlu, modulates the effects of the neurotoxin, 1-methyl-4-phenyl-1,2,3,6-tetrahydropyridine (MPTP), on behavioral and cognitive performance in female and male mice. We studied whether these effects are associated with changes in striatal tyrosine hydroxylase (TH) levels and the gut microbiome. Two-week sub-chronic MPTP increased activity of female and male wild-type (WT) and mGlu8 knockout (KO) mice in the open field. MPTP also showed genotype- and sex-dependent effects. MPTP increased the time WT, but not KO, females and males spent exploring objects. In WT mice, MPTP improved sensorimotor function in males but impaired it in females. Further, MPTP impaired cued fear memory in WT, but not KO, male mice. MPTP reduced striatal TH levels in WT and KO mice but these effects were only pronounced in males. MPTP treatment and genotype affected the diversity of the gut microbiome. In addition, there were significant associations between microbiome α-diversity and sensorimotor performance, as well as microbiome composition and fear learning. These results indicate that specific taxa may directly affect motor and fear learning or that the same physiological effects that enhance both forms of learning also alter diversity of the gut microbiome. MPTP’s effect on motor and cognitive performance may then be, at least in part, be mediated by the gut microbiome. These data also support mGlu8 as a novel therapeutic target for PD and highlight the importance of including both sexes in preclinical studies.

## Introduction

Parkinson’s disease (PD) results in motor symptoms that have largely been attributed to the loss of substantia nigra dopaminergic neurons (Damier et al., 1999; Björklund and Dunnett, 2007). Current treatment options focus on targeting these motor deficits by supplementing dopamine with its precursor, levodopa. Long-term treatment with levodopa often results in patients becoming tolerant and developing dyskinesia. There are currently no therapies that alter disease progression, highlighting a need for more preclinical research.

PD also manifests with non-motor symptoms, including cognitive impairments, depression, and alterations in circadian rhythm. These non-motor symptoms are particularly important during early PD and prior to the initiation of dopaminergic treatment because they are more strongly linked to reduced quality of life than motor symptoms (Müller et al., 2013). Depression is a risk factor for developing PD and associated with cognitive impairment possibly involved with abnormal regulation of glutamate (Kashani et al., 2007; Sanacora et al., 2012; Shen et al., 2013; Emre, 2015). In addition, chronic lack of sleep and irregular sleep-wake cycle, including falling asleep, staying asleep, and excessive daytime sleepiness, are increasingly reported before the onset of PD suggesting that they could be risk factors or part of prodromal PD (Abbot et al., 2005; Chen et al., 2006; Gao et al., 2011; Breen et al., 2014).

Many PD patients also present with digestive symptoms, including constipation, years before they have neurological symptoms. In addition, patients show increased intestinal permeability, or leaky gut, that can lead to the translocation of bacteria and bacteria-related inflammatory products (Forsyth et al., 2011). One study found differences in taxa abundance in the gut but not nasal microbiome between PD patients and controls (Heintz-Buschart et al., 2018). Alterations in the gut microbiome have been proposed as the earliest event in PD pathogenesis. Gut microbiota have been shown to regulate motor impairments and neuroinflammation in α-synuclein mice (Sampson et al., 2016). The gut microbiome can communicate with the CNS via the gut-brain axis, and affect stress-related behaviors, anxiety and depression (Foster and McVey Neufeld, 2013; Kelly et al., 2015; Allen et al., 2017).

Both the motor and non-motor symptoms have been shown to be sex-dependent. While men are more likely to develop PD than women, women tend to present with more severely affected anxiety, nutritional status and quality of life scores (Miller and Cronin-Golomb, 2010; Farhadi et al., 2017). Interestingly, dopamine loss may be causative for such emotion-related symptoms and occur independent of motor deficits (Drui et al., 2014). Sleep disturbances seen in PD patients also appear to be sex-dependent, with men demonstrating a higher prevalence of sleep disorders than women (Ozekmekçi et al., 2005; Yoritaka et al., 2009).

Consistent with these human data, disturbances in circadian rhythms are seen prior to the onset of PD symptoms and worsen motor and cognitive deficits in a PD mouse model involving exposure to the neurotoxin 1-methyl-4-phenyl-1,2,3,6-tetrahydropyridine (MPTP; Lauretti et al., 2017). MPTP is converted into 1-methyl-4-phenylpyridinium (MPP^+^) in glia and taken up by DA transporters on presynaptic dopaminergic nerve terminals. MPP^+^ not sequestered into vesicles is taken up by mitochondria and disrupts oxidative phosphorylation resulting in bioenergetic failure and cell death (Przedborski and Jackson-Lewis, 1998). The MPTP model has increased the understanding of the pathophysiology of PD and serves as a critical model to test therapeutic interventions (for a review see Bové and Perier, 2012).

Glutamatergic neurotransmission is perturbed in many neuropathological conditions. L-glutamate, the major excitatory neurotransmitter (Coyle, 2006), activates both ionotropic and metabotropic glutamate receptors (mGlu). The mGlu, a family of G protein-coupled receptors, are divided into three groups on the basis of sequence homology, signal transduction mechanisms, and pharmacologic properties (Pin and Duvoisin, 1995). The mGlu III family includes mGlu 4, 6, 7 and 8 and negatively regulate adenylyl cyclase via G_αi_. Except for mGlu6, they are mainly localized presynaptically and can act as autoreceptors to inhibit glutamate release. PD patients show cognitive problems that become apparent as PD progresses and a 50% decrease in levels of vesicular glutamate transporter-1 (VGLUT-1) in the prefrontal cortex (Kashani et al., 2007; Emre, 2015). This decrease in VGLUT-1 likely causes reduced glutamate transmission.

Currently, mGlu4 agonists and other interventions are being evaluated for treating motor function in PD (Lopez et al., 2007; Rylander et al., 2010; Lindsley and Hopkins, 2012; Almaric et al., 2013) but these are not anticipated to have beneficial effects on cognition. The mGlu8 agonist (*S*)-3,4-dicarboxyphenylglycine (DCPG) evoked catalepsy, a PD symptom, and enhanced catalepsy induced by haloperidol (Ossowska et al., 2007). Therefore, mGlu8 inhibition or deficiency might be beneficial to improve motor function in PD. mGlu8 inhibition might reduce depressive behaviors as well, since mGlu8 has been implicated in depression (Terracciano et al., 2011). The protective effects of mGlu8 might not be limited to MPTP and PD but also extend to other neurodegenerative conditions. Increased expression of mGlu8 in microglia was suggested in patients with sporadic Creutzfeldt-Jakob disease with a risk allele containing a GRM8 genetic variant (Sanchez-Juan et al., 2015).

The goal of this study was to determine if mGlu8 deficiency might protect against the sub-chronic effects of MPTP in female and male mice and whether these effects are associated with changes in tyrosine hydroxylase (TH) levels in the striatum as well as alterations in the gut microbiome. We used a 2-week MPTP exposure regimen as the 4-week MPTP exposure regimen previously used to model PD symptoms (Goldberg et al., 2011) resulted in motor impairments too severe for assessment of behavioral and cognitive performance. Specific behaviors looked at included locomotion, object recognition, fear learning, spatial memory, depressive-like behavior and circadian activity.

## Materials and Methods

### Animals

**Figure 1:**
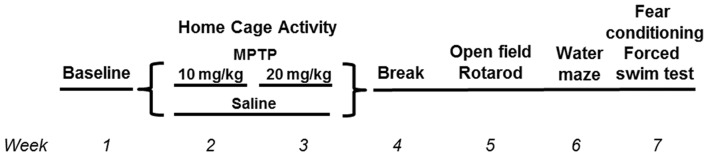
Time line of behavioral and cognitive tests.

This study was carried out in accordance with the recommendations of “ARRIVE guidelines.” The protocol was approved by the Institutional Animal Care and Use Committee at OHSU and followed the ARRIVE guidelines. C57BL/6J wild-type (WT) and mGlu8 knockout (KO) mice (Duvoisin et al., 2005) were used for this study. The mice were group housed until the start of the assessment of circadian activity, 1 week prior to the first MPTP or saline treatment, at which point they were then singly housed. All mice had access to food (PicoLab Rodent Diet 20, no. 5053; PMI Nutrition International, St. Louis, MO, USA) and water *ad libitum* throughout the study. Housing facilities were maintained at 20–21°C and kept on a constant 12 h light:12 h dark cycle. Mice were monitored for general health throughout the study. See Figure 1 for the experimental timeline and Table 1 for group number breakdowns.

**Table 1:** Group sizes in this study^1^.

	WT		KO	
	Females	Males	Females	Males
Saline	14 (5, 2)	14 (12, 2)	11 (5, 2)	11 (11, 3)
MPTP	10 (5, 2)	13 (12, 3)	12 (7, 4)	13 (14, 2)

*^1^Numbers in parentheses reflect the subset of mice used for activity monitoring and gut microbiome data, respectively*.

### Treatment and Home Cage Activity Monitoring

MPTP (Santa Cruz Biotechnology), dissolved in saline and measured as the base, or saline was administered via intraperitoneal injections daily for 10 days, total over 2 weeks during the light period, between 9 am and 9:30 am (lights on at 6:30 am). All mice were treated at the same time each day, and different researchers treated and behaviorally tested the mice. The first 5 days of week 1 consisted of a 10 mg/kg MPTP dosage, followed by a 2-day break, then followed by a 20 mg/kg MPTP dosage for 5 days of week 2. Control animals were administered saline (Figure 1).

During the 2 weeks of treatment, circadian home cage activity of the mice was continuously monitored using an unobtrusive home cage sensor system MLog (BioBServe, Germany) and a conventional Metro rack cart. Only a subset of mice was included for this analysis. The mice that were not included for home cage activity were housed under similar conditions on a ventilated cage rack. Data were recorded from each monitor every second and were averaged across 30-min bins for analysis.

### Behavioral and Cognitive Testing

Mice were tested for behavioral and cognitive changes using the following test order (see Figure 1): sensorimotor function on the rotarod, exploratory activity and measures of anxiety in the open field for three subsequent days and object recognition for two subsequent days, spatial learning and memory in the Morris water maze, emotional learning and memory in the contextual and cued fear conditioning tests, and for depressive-like behavior in the forced swim test. The experimenter was kept blind to both genotype and treatment throughout the testing.

### Open Field and Object Recognition

General locomotion and anxiety-like behavior was measured in an open environment was assessed using an open square arena (40.6 cm in length) with transparent walls. Animals were placed into the maze for a single 5-min trial in the morning for 3 days in a row. On the subsequent 2 days, mice were placed again in the open field containing two small blocks for a 15-min trial per day to assess object recognition. The light intensity within the maze ranged from 300 lux to 500 lux. Movement of the mice was recorded using video tracking with Ethovision XT 7 software (Noldus Information Technologies, Wageningen, Netherlands). Dependent measures recorded were distance moved and time spent exploring the objects (defined as the nose within 1 cm of the objects).

### Rotarod

Sensorimotor function was assessed using the rotarod. Mice were placed on a rotating rod (diameter: 3 cm, elevated: 45 cm; Rotamex-5, Columbus Instruments, Columbus, OH, USA). Rotation speed started at 5.0 rpm and accelerated 1.0 rpm every 3 s. Latency to fall (s) was recorded using photo beams located below the rod. Mice received three trials each day for three subsequent days.

### Water Maze

Spatial learning and memory requiring navigation was assessed using the Morris water maze (Johnson et al., 2015). Mice were first trained to locate an escape platform marked with a visible flag on the first day. The next day, they were trained to locate the first hidden platform location using spatial cues. After 2 days of training, the hidden platform was moved to a new location. Each hidden platform location was followed by a test for spatial memory retention using a 1-min probe trial. Latency to reach the platform, swim speed, cumulative distance to the target location, and percent time spent in each quadrant of the pool were analyzed using Ethovision XT7 Software. The water maze data were not included in this publication.

### Fear Conditioning

Fear conditioning was used to assess hippocampus- and amygdala-dependent associative memory (Anagnostaras et al., 2010). Mice were trained to associate a mild foot shock (unconditioned stimulus) with a short tone (80 dB; conditioned stimulus) in a sound-attenuated chamber with wire floors (Med Associates Inc., St. Albans, VT, USA). During training, mice were placed in a chamber and allowed to explore it during a 2-min baseline period. They were then exposed to two sets of 30-s tones, each co-terminating with a 2-s foot shock (0.5 mA). The next day hippocampal-dependent contextual fear memory was assessed by placing the mice back in chambers for a 5-min trial without tones or shocks. During training and the contextual fear memory test, the chambers were cleaned with 0.5% acetic acid. Four hours after the contextual fear memory test, amygdala-dependent cued memory was assessed by placing the mice in chambers that were distinguished from the prior context using flat floor inserts, a triangular ceiling, and a new odor (vanilla extract). During this test for cued memory, mice were exposed to a 2-min baseline after which a 3-min tone was played. During assessments of cued fear memory, the chambers were cleaned with 70% ethanol between the trials.

Average motion (cm/s) and time spent freezing were analyzed using Video Freeze software (Med Associates, VT, USA). Freezing was defined as no movement except respiration for a minimum duration of 30 frames.

### Forced Swim Test

Mice were assessed for depressive-like behavior using the forced swim test. Groups of four mice were individually placed into separate 2 L glass beakers (diameter = 12.7 cm) and allowed to swim during one 6-min trial. Water in the beakers was kept at room temperature (21°C). Videos were recorded and manually scored for total time immobile for the last 5 min during the forced swim task, as described (McGinnis et al., 2017).

### Western Blot analysis

Striatal brain tissues were homogenized in RIPA lysis buffer (0.1% SDS, 0.5% sodium deoxycholate, 1% NP-40, 150 mM NaCl, 50 mM Tris pH 8.0) containing the phosphatase inhibitor sodium vanadate (NaV, 1 mM). Protein lysates were extracted by centrifugation and protein concentrations were determined in duplicate samples using the Micro BCA protein assay. Equal amounts of protein were separated on sodium dodecyl sulfate polyacrylamide (4%–12% SDS-PAGE) gradient gels and transferred to a polyvinylidene difluoride (PVDF) membrane. Membrane blots were then blocked in tris-buffered saline and tween 20 (TBST; 1× TBS, 0.1% Tween 20) containing 5% milk. The membranes were then incubated with anti-TH primary polyclonal antibody (1:1000; Millipore) diluted in TBST for 90 min at room temperature. Membranes were then washed in TBST (3 × 10 min) before being incubated in donkey anti-rabbit secondary antibody (1:10,000 dilution) for 1 h at room temperature. Membranes were incubated in enhanced chemiluminescence (ECL) reagent before being exposed to CL-XPosure Film to detect protein changes. Membranes were then washed in stripping solution (12 g urea, 1.7 ml 1.5 M NaCl, 1.7 ml 0.5 M EDTA, di H_2_O) for 1 h to strip antibodies bound. Membranes were then probed with β-actin primary monoclonal mouse antibody (1:10,000; Santa Cruz) diluted in TBST for 60 min at room temperature. Next, membranes were washed in TBST (3 × 10 min) before being incubated in donkey anti-mouse secondary antibody (1:10,000 dilution) for 1 h. Membranes were incubated in ECL reagent before being exposed to CL-XPosure Film to detect changes in TH and β-actin protein.

### Microbiome Sequencing

Bacterial 16S rDNA sequences were extracted and sequenced as previously described (Gaulke et al., 2016). Briefly, DNA was extracted from collected fecal pellets using the QIAgen DNeasy Power Soil kit (Qiagen, Hilden, Germany) following the manufacture’s protocol with the addition of an incubation step of 10 min at 65°C before bead beating. Extracted DNA was amplified using the Earth Microbiome Project 16S PCR protocol, targeting the V4 region of the 16S rDNA gene; PCR reactions were conducted in triplicate for each sample. The amplicons were run on a 1% agarose gel for quality control, the reactions were cleaned with the UltraClean PCR clean-up kit (MOBIO, Carlsbad, CA USA), and diluted to produce 200 ng of DNA per sample. The prepared libraries were submitted to the Oregon State University Center for Genome Research and Biocomputing for 250 bp paired-end sequencing on an Illumina MiSeq instrument. Due to poor quality of the reverse reads from the MiSeq, we used only the forward reads, trimmed to 240 bp. Quality control, exact sequence variants clustering and chimera removal were conducted using the dada2 package (Callahan et al., 2016) for R (R Core Team 2017). Dada2 also assigned taxonomy to the sequence variants utilizing the Silva taxonomic training data formatted for dada2. Exact sequence variants were aligned using mafft (Katch et al., 2002) and a phylogenetic tree of the bacterial community from all samples was generated using FastTree (Price et al., 2009). Sequences were rarefied to 50,846 sequences per sample.

### Statistical Analyses

Behavioral and Western blot data are reported as mean ± standard error of the mean and were analyzed using SPSS v.22 software (IBM, Armonk, NY, USA). Graphs were generated using GraphPad software (La Jolla, CA, USA). As we have previously shown that mGlu8 KO mice demonstrate altered behavioral phenotypes—i.e., reduced exploratory behavior and contextual fear memory and increased anxiety-like behavior—and furthermore* a priori* hypothesized that WT and KO mice would differently respond to MPTP treatment, WT and KO were analyzed separately. Sex and treatment group were included as factors in 2-way analysis of variance (ANOVAs). Repeated-measures were used when appropriate as indicated. Statistical significance was considered as *p* < 0.05. When sphericity was violated (Mauchly’s test), Greenhouse-Geisser corrections were used. The mice were tested in three separate cohorts. Cohort was included as a factor initially during analysis but was removed as it was found to be not significant.

For statistical analysis of the microbiome data, we estimated α-diversity, diversity within samples, using the Shannon index, Simpson index, Chao1 and phylogenetic diversity. The best model for predicting α-diversity was selected by using stepwise Aikake Information Criterion (AIC), and significant associations between α-diversity and the variables in the selected model were assessed using a standard ANOVA model. We estimated β-diversity, diversity between samples, using the Bray-Curtis dissimilarity, and produced an ordination using principal coordinate analysis (PCoA). The best model for predicting microbiome composite was selected using a permutational-ANOVA (PERMANOVA) analog to stepwise AIC implemented by the function ordistep from the vegan package in R (Oksanen et al., 2018). A multiple correlation test with false discovery rate correction on pairwise *t*-tests was performed to determine significant associations between individual taxa and covariates of interest. A hierarchical linear discriminate analysis, LefSe (Segata et al., 2011), was used to determine taxa that were indicative of MPTP treatment vs. controls.

## Results

### Circadian Home Cage Activity

**Figure 2:**
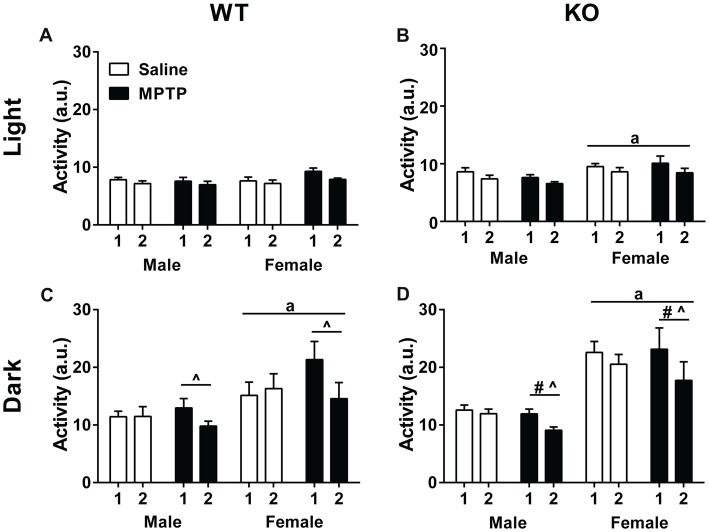
Home cage activity of wild-type (WT; **A,C**) and knockout (KO; **B,D**) during the light **(A,B**) and dark **(C,D)** periods during the treatment period. **(A,B)** WT and KO mice showed less activity during the second week of treatment (WT: *p* < 0.001; KO: *p* < 0.001). KO female mice moved more during the light cycle than KO males. ^a^*p* = 0.019. **(C,D)** During the dark cycle, WT and KO mice showed a similar decrease in activity during week 2 (WT: *p* = 0.004; KO: *p* < 0.001) that was affected by 1-methyl-4-phenyl-1,2,3,6-tetrahydropyridine (MPTP) treatment. WT: ^^^*p* < 0.001; KO: ^^^*p* = 0.001. WT and KO females moved more than males; WT: ^a^*p* = 0.005; KO: ^a^*p* < 0.001. KO females also moved less during week 2 than KO males. ^#^*p* = 0.015.

Home cage activity was analyzed using a repeated-measures ANOVA for average activity during the light and dark cycles during the 2 weeks of treatment. Analysis of activity during the light phase showed higher levels of activity of WT mice in week 1 compared to week 2 (*F*_(1,30)_ = 28.650, *p* < 0.001) but there were no significant effects due to sex or treatment (Figure 2A). Similarly, KO mice showed higher activity levels during the light phase in week 1 than week 2 (*F*_(1,34)_ = 32.639, *p* < 0.001). In addition, KO females showed greater activity levels than KO males (*F*_(1,34)_ = 6.036, *p* = 0.019) but there was no effect of treatment (Figure 2B).

Analysis of activity levels during the dark phase also revealed higher activity levels in week 1 than week 2 in both genotypes (WT: *F*_(1,30)_ = 9.814, *p* = 0.004; KO: *F*_(1,34)_ = 32.639, *p* < 0.001). In addition, there was an interaction between week and treatment (*F*_(1,30)_ = 16.015, *p* < 0.001; KO: *F*_(1,34)_ = 13.012, *p* = 0.001). When the two treatment groups were analyzed separately, there was only an effect of week in the MPTP group. MPTP increased activity in week 1, but not week 2.

Further, sex was a significant factor for activity levels in the dark phase for both genotypes with females moving more than males (WT: *F*_(1,30)_ = 9.220, *p* = 0.005; KO: *F*_(1,34)_ = 36.469, *p* < 0.001). In KO mice, there was also an interaction between week and sex (*F*_(1,34)_ = 13.012, *p* = 0.001; Figures 2C,D).

### Open Field and Object Recognition

**Figure 3:**
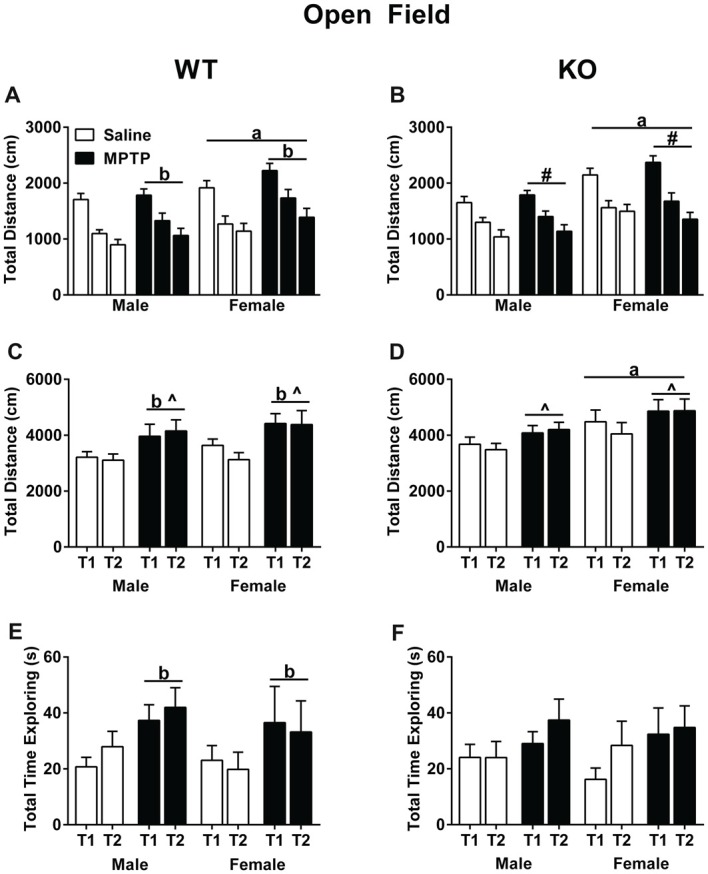
Activity of saline- and MPTP-treated WT and KO mice in the open field **(A,B)**, in the open field containing two objects over two subsequent days **(C,D)**, and total time spent exploring the objects **(E,F)**. **(A,B)** All groups habituated to open field; effect of day; WT: *p* < 0.001; KO: *p* < 0.001. WT mice treated with MPTP traveled more distance than saline-treated WT mice. ^b^*p* = 0.043. WT and KO females moved more than genotype-matched males in the open field. WT: ^a^*p* = 0.001; KO: ^a^*p* < 0.001. **(C,D)** WT MPTP-treated mice moved more than saline mice in the open field containing two objects (*p* = 0.004). WT and KO saline-treated mice moved less on the second day than the first day in the open field containing two objects. WT: ^^^*p* = 0.024; KO: ^^^*p* = 0.04. KO females moved more overall than KO males. ^a^*p* = 0.038). **(E,F)** In WT mice, MPTP increased the time the mice spent exploring the objects. ^b^*p* = 0.034. ^#^ refers to an interaction between sex and day (*F*_(2,84)_ = 3.880; *p* = 0.024).

Total distance traveled was analyzed using a repeated-measures ANOVA for each of the three exposures to the open field without the objects as well as the two exposures to the open field containing the objects. In WT mice, activity levels decreased over the 3 days (Mauchly’s test of sphericity: *p* = 0.047, Greenhouse-Geisser corrected, *F*_(1.747,69.860)_ = 100.797; *p* < 0.001, Figure 3A), indicating that the mice recognized the environment and habituated to it. In addition, WT females moved more than males (*F*_(1,40)_ = 5.900; *p* = 0.001). Strikingly, MPTP-treated mice moved more than saline-treated mice (*F*_(1,40)_ = 4.368; *p* = 0.043). Similar as in WT mice, KO mice habituated to the open field and showed decreased activity levels over the 3 days in total distance traveled (*F*_(2,84)_ = 92.646; *p* < 0.001, Figure 3B). There was also a significant interaction between sex and day (*F*_(2,84)_ = 3.880; *p* = 0.024) and KO females traveled more than KO males (*F*_(1,42)_ = 15.610; *p* < 0.001).

When exposed to objects in the open field, both saline-treated WT (Figure 3C) and KO (Figure 3D) mice moved less during the second exposure to the objects, which was not seen in the MPTP-treated groups (WT: *F*_(2,84)_ = 5.408; *p* = 0.024; KO: *F*_(1,43)_ = 4.484; *p* = 0.040). Furthermore, WT MPTP-treated mice moved significantly more than WT saline-treated mice (*F*_(1,47)_ = 9.311; *p* = 0.004). This treatment effect was genotype-dependent and not seen in KO mice. In KO mice, females moved more in the presence of the objects than males (*F*_(1,43)_ = 4.597; *p* = 0.038). Analyses of the total time spent exploring the objects showed that in WT mice MPTP-treated mice explored the objects more than saline-treated mice (*F*_(1,47)_ = 4.747; *p* = 0.034, Figure 3E). This treatment effect was not seen in the KO mice (Figure 3F).

### Rotarod Performance

**Figure 4:**
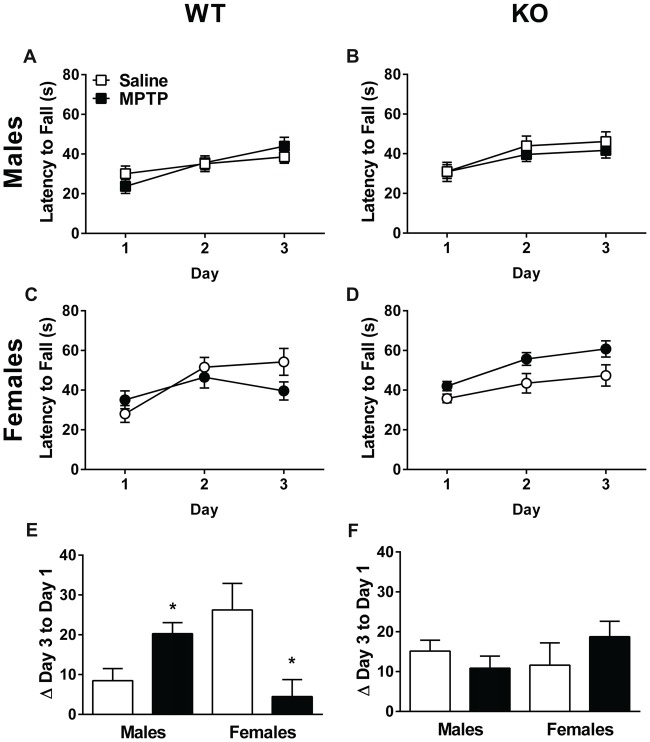
Rotarod performance of saline- and MPTP-treated WT **(A,C,E)** and KO **(B,D,F)** mice. The latency to fall is shown in **(A–D)** and the change from the average latency to fall on Day 1 to the average latency to fall on Day 3 in **(E,F)**. WT **(A,C)** and KO **(B,D)** mice showed improvement on the rotarod over the 3 days of testing. WT: *p* < 0.001; KO: *p* < 0.001. Females of both genotypes performed better than males. WT: *p* = 0.049; KO: *p* = 0.018. **(E,F)** In WT mice, MPTP-treated males improved more than saline-treated males, while MPTP-treated females performed more poorly than saline-treated females. *p = 0.001. This effect was genotype-dependent and not seen in KO mice. The * refers to a sex × treatment interaction: WT-Change score from day 3 average to day 1 average; sex × treatment interaction: *F*_(1,47)_ = 13.162, *p* = 0.001.

Next, sensorimotor performance on the rotarod was assessed. The learning curves of the WT and KO mice are shown in Figures 4A–D. Both genotypes showed an improvement in performance on the rotarod with training (Repeated-measures ANOVA; WT: Mauchly’s test of sphericity: *p* = 0.015, Greenhouse-Geisser corrected, *F*_(1.713,80.510)_ = 34.123, *p* < 0.001; KO: *F*_(2,86)_ = 33.318, *p* < 0.001). In addition, females performed better than males and showed longer latencies to fall regardless of treatment or genotype (WT: *F*_(1,47)_ = 4.083, *p* = 0.049; KO: *F*_(1,43)_ = 6.098, *p* = 0.018). In WT mice, there was a day by sex interaction (*F*_(2,94)_ = 3.167, *p* = 0.047), as well as a day by sex by treatment interaction (*F*_(2,94)_ = 9.243, *p* < 0.001, Figures 4A,C). In KO mice, there was a trend towards a sex by treatment interaction but it did not reach significance (*F*_(1,43)_ = 3.811, *p* = 0.057, Figures 4B,D).

The improvement in performance with training was also assessed by calculating the difference between Day 3 and Day 1’s average latency to fall. A positive difference implies improved performance. Analysis of the change between the average latency to fall on Day 1 to Day 3 revealed a significant sex × treatment in WT mice (*F*_(1,47)_ = 13.162, *p* = 0.001, Figure 4E). Specifically, MPTP-treated males performed better than saline-treated males whereas MPTP-treated females performed more poorly than saline-treated females. This was not seen in KO mice (Figure 4F).

### Fear Learning and Memory

**Figure 5:**
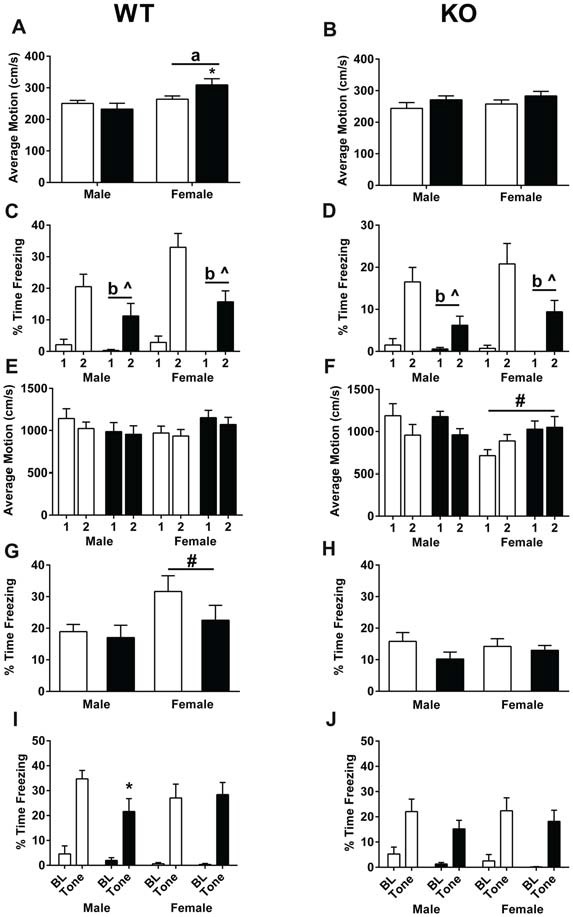
Fear learning and memory of saline- and MPTP-treated WT **(A,C,E,G,I)** and KO **(B,D,F,H,J)** mice. **(A)** In WT mice, females showed higher activity than males during the baseline period (prior to the first tone). ^a^*p* = 0.003. In addition, MPTP increased activity levels in females (*p = 0.035) but not males. **(B)** No sex or MPTP treatment effects were seen in KO mice. **(C,D)** WT and KO mice demonstrated learning of the tone-shock pairing, indicated by the higher time spent freezing during tone 2 compared to tone 1. WT: *p* < 0.001; KO: *p* < 0.001. Furthermore, MPTP treatment in both genotypes resulted in less freezing during the tone (WT: ^b^*p* = 0.001; KO: ^b^*p* = 0.007) as well less of an increase in freezing going from tone 1 to tone 2 (WT: ^^^*p* = 0.0012; KO: ^^^*p* = 0.007). **(E)** This was not due to a differential response to the shock in WT mice. **(F)** In KO mice, females did not show a similar decrease in average motion during shock 2 compared to shock 1 as seen in males. ^#^*p* < 0.001. **(G)** During the contextual fear memory test, WT females spent more time freezing than WT males. ^a^*p* = 0.031. **(H)** No sex differences in contextual fear memory were seen in KO mice. **(I)** In WT mice, MPTP impaired cued fear memory in males, but not females. *p = 0.0415. **(J)** MPTP did not affect cued fear memory in KO male or female mice. ^^^ refers to interactions between treatment and tone; b refers to a significant treatment effect.

During training, average motion (cm/s) was assessed during the 90-s baseline. In WT mice, there was a sex × treatment interaction (*F*_(1,47)_ = 4.732, *p* = 0.035, Figure 5A). WT females treated with MPTP moved more than WT females treated with saline (*p* < 0.05, Figure 5A). In addition, WT females moved more than WT males (*F*_(1,47)_ = 9.479, *p* = 0.003, Figure 5A). In contrast, there were no significant effects of sex or treatment in the KO mice (Figure 5B). Analysis of the percent time spent freezing during each of the tones during training showed that while both genotypes learned, indicated by the higher percent time spent freezing during the second tone compared to the first tone (WT: *F*_(1,47)_ = 79.550, *p* < 0.001; KO: *F*_(1,43)_ = 50.583, *p* < 0.001, Figures 5C,D), WT and KO MPTP-treated mice showed less time spent freezing during tone 2 compared to tone 1 than saline-treated mice (repeated-measure ANOVA; WT: *F*_(1,47)_ = 6.761, *p* = 0.012; KO: *F*_(1,43)_ = 8.056, *p* = 0.007, Figures 5C,D). Overall, MPTP-treated mice displayed less time spent freezing during the tones (RM ANOVA; WT: *F*_(1,47)_ = 11.905, *p* = 0.001; KO: *F*_(1,43)_ = 12.140, *p* = 0.001, Figures 5C,D). When response to the shocks was analyzed, there were no effects of sex or treatment in WT mice (repeated-measures ANOVA, Figure 5E). However, in KO mice, there was a shock × sex interaction (repeated-measures ANOVA; *F*_(1,43)_ = 14.928, *p* < 0.001, Figure 5F). Males had lower average motion during the second shock which was not seen in females.

Mice were then tested for contextual fear memory. MPTP did not significant affect contextual fear memory in WT or KO mice. WT female mice froze more that WT male mice (*F*_(1,47)_ = 4.963, *p* = 0.031). When cued fear memory was assessed, both WT and KO mice froze more during the tone than prior to the tone (repeated-measures ANOVA; WT: *F*_(1,47)_ = 126.732, *p* < 0.001; KO: *F*_(1,43)_ = 79.207, *p* < 0.001, Figures 5G,H). In WT males, mice treated with MPTP froze less during the tone than mice treated with saline (unpaired two-tailed *t*-test, *p* = 0.0415, Figure 5I). This was genotype- and sex-dependent and not seen in WT females (Figure 5I) or KO males or females (Figure 5J).

### Striatal Levels of Tyrosine Hydroxylase and β-Actin

**Figure 6:**
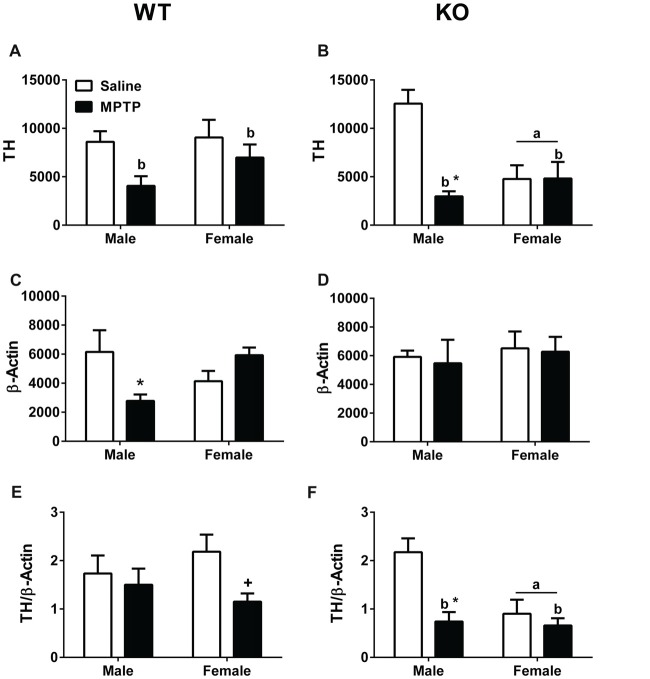
Western blot analysis of tyrosine hydroxylase (TH) and β-actin levels in striatum of saline- and MPTP-treated WT and KO mice. **(A,B)** WT and KO mice treated with MPTP showed lower TH levels than saline-treated mice. WT: ^b^*p* = 0.020; KO: ^b^*p* = 0.003. In addition, in KO mice, females showed lower TH levels than males. ^a^*p* = 0.049. Further, KO males treated with MPTP showed lower TH levels compared to saline-treated KO male mice. *p = 0.003. This MPTP-dependent decrease was sex-dependent and not seen in KO females. **(C)** WT males showed an MPTP-dependent decrease in β-actin levels (*p = 0.011). This effect was sex-dependent and not seen in WT females. **(D)** MPTP did not affect β-actin levels in KO mice. **(E,F)** When TH and β-actin levels were analyzed as a ratio, KO females showed lower TH/β-actin ratios. ^a^*p* = 0.011. In addition, KO MPTP-treated mice showed lower ratios than saline-treated KO mice. ^b^*p* = 0.001. These treatment effects seemed driven by a MPTP-dependent decrease in KO males (*p = 0.023) not seen in KO females. In WT females, there was a trend towards lower TH/β-actin ratios in MPTP-treated females but this did not reach significance. ^+^*p* = 0.065.

Striatal (dorsolateral) brain tissues were analyzed for TH and β-actin levels. TH levels (Figures 6A,B), β-actin levels (Figures 6C,D), and the TH/β-actin ratios (Figures 6E,F) are shown. TH levels were lower in WT mice treated with MPTP than WT mice treated with saline (*F*_(1,22)_ = 6.254; *p* = 0.020, Figure 6A). Similarly, TH levels were lower in KO mice treated with MPTP than those treated with saline (*F*_(1,18)_ = 11.776; *p* = 0.003, Figure 6B). In addition, in KO mice the TH levels were lower in females than males (*F*_(1,18)_ = 4.465; *p* = 0.049, Figure 6B) and there was a sex × treatment interaction (*F*_(1,18)_ = 11.776; *p* = 0.003, Figure 6B). TH levels were lower in male mice treated with MPTP than male mice treated with saline; this was not seen in female mice.

In WT male mice, β-actin levels were lower in MPTP-treated mice compared to saline-treated males (*F*_(1,22)_ = 7.619; *p* = 0.011; *post hoc* *t*-test between male treatment groups: *p* = 0.05). This was not seen in WT female mice, which showed a trend towards an increase in β-actin levels following MPTP treatment than following saline treatment (*post hoc* *t*-test between female treatment groups: *p* = 0.070). No effect of MPTP was seen in female or male KO mice.

When the ratio between TH and β-actin was analyzed, there was a trend towards lower TH/β-actin ratios in WT mice treated with MPTP than WT mice treated with saline (*p* = 0.065, Figure 6E). This trend was driven by the lower TH/β-actin ratio in WT female mice treated with MPTP than those treated with saline (*t*-test, *p* = 0.024, Figure 6E). In KO mice, the TH/β-actin ratios were lower in MPTP-treated mice than saline-treated mice (*F*_(1,18)_ = 12.370; *p* = 0.001, Figure 6F). In addition, KO MPTP-treated males showed lower TH/β-actin ratios than saline-treated males (*F*_(1,18)_ = 6.228; *p* = 0.023, Figure 6F). This treatment effect was sex-dependent and not seen in females. Finally, KO females showed lower TH/β-actin ratios than KO males (*F*_(1,18)_ = 8.081; *p* = 0.011, Figure 6F).

### Microbiome

Bacterial DNA from mouse fecal pellets was extracted, and the 16S rDNA gene was amplified and sequenced to determine the diversity and composition of the intestinal microbiome. The number and abundance of bacterial taxa (α-diversity) found in each fecal sample were quantified to determine if there were significant associations between diversity and various experimental covariates.

**Figure 7:**
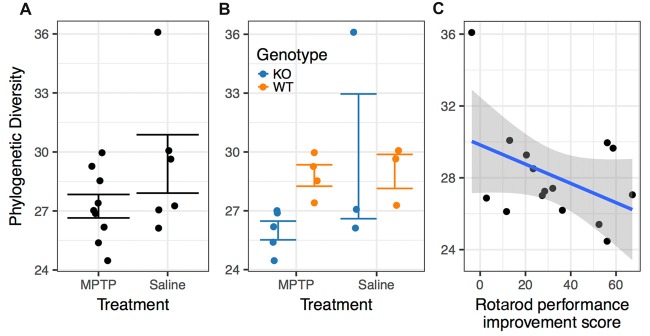
Associations between phylogenetic diversity. **(A)** The phylogenetic diversity of the microbiomes associated with MPTP-mice was significantly lower than that of saline-treated mice (*p* = 0.009). **(B)** Differences between genotypes within the MPTP group that are not significant in the saline-treated group. **(C)** Rotarod performance improvement score significantly associated with α-diversity (*p* = 0.001).

There was no significant difference in microbiome α-diversity between the MPTP-treated and saline-treated mice for any of the taxonomic measures (Chao1, Shannon, or Simpson) we assessed. However, the phylogenetic diversity of the microbiomes associated with MPTP-mice was significantly lower than that of saline-treated mice (*F*_(1,4)_ = 22.774; *p* = 0.009 Figure 7A, Table 2). These results indicate that while MPTP-treatment does not affect the number of taxa present in the mouse gut microbiome, it does result in a microbiome that is comprised of more closely related organisms. That said, this observation could be driven by the single mouse in the saline-treated group with a particularly high phylogenetic diversity.

**Table 2:** Analysis of variance (ANOVA) of best model (as determined by aikake information criterion (AIC)) for prediction of phylogenetic diversity by experimental covariates.

	Df	SumSq	MeanSq	*F* value	Pr(>F)^1^
MPTP treatment	1	16.638	16.638	22.773	**0.009**
Genotype	1	6.920	6.920	9.471	**0.037**
as.factor (DOB)	3	17.246	5.749	7.868	**0.037**
Home cage activity during Week 1 (dark cycle)	1	1.029	1.029	1.408	0.301
Total distance moved during object introduction in the open field	1	1.803	1.803	2.468	0.191
Rotarod performance improvement score	1	48.966	48.966	67.018	**0.001**
Time freezing during tone in cued memory test	1	2.777	2.777	3.801	0.123
MPTP treatment:genotype	1	10.023	10.023	13.718	**0.021**
Residuals	4	2.923	0.731		

*^1^Significant *p* values are indicated in bold*.

The two genotypes showed a significance difference in α-diversity, with WT mice microbiomes manifesting greater diversity than KO mice microbiomes (*F*_(1,4)_ = 9.471; *p* = 0.037, Table 2). Furthermore, we observed a significant interaction between genotype and treatment group (*F*_(1,4)_ = 13.718; *p* = 0.021, Table 2), indicating that the effect of genotype on α-diversity differs between MPTP-treated and saline-treated mice. In particular, the difference in α-diversity between genotypes appeared to be driven primarily by differences between genotypes within the MPTP group that are not significant in the saline-treated group (Figure 7B).

**Figure 8:**
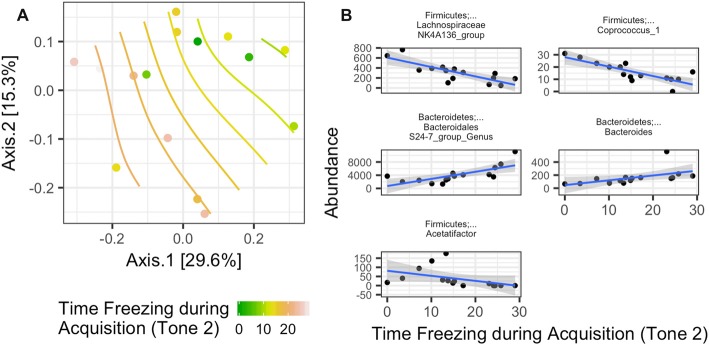
**(A)** Significant association between the microbiome composition (Bray-Curtis dissimilarity matrix) and the amount of time spent freezing during the second tone, a measure of fear learning, in the fear conditioning test (Time Freezing during acquisition (Tone 2; *p* = 0.028). **(B)** This was driven by five taxon abundances that significantly correlate with the amount of time spent freezing after the second tone in the fear learning and memory test.

Rotarod performance improvement score, reflecting the improvement in the rotarod test based on performance in the 9th trial as compared to the 1st trial, significantly associated with α-diversity (*F*_(1,4)_ = 67.018; *p* = 0.001, Figure 7C, Table 2). Mice with more improvement in rotarod test performance had lower microbiome diversity, though the evenness or how individuals were distributed amongst the different species of the microbiome remained relatively the same (Simpson metric). There was also a significant negative relationship between the Chao1 metric and Time Freezing during Acquisition (Tone 2), reflecting the percent time the mice froze during the second tone of the fear conditioning training after having experienced the first tone co-terminating with a foot shock (*F*_(1,4)_ = 150.230; *p* < 0.001, Supplementary Table S1). This also indicates that mice that learned to acquire fear better had lower numbers of microbial taxa, though this term was not kept in the model by AIC selection for phylogenetic diversity. Additionally, the composition of the microbiome varied significantly with Time Freezing during Acquisition (Tone 2; *F*_(1,13)_ = 1.97; *p* = 0.028, Figure 8A), primary driven by decreases in a three Firmicutes OTUs (the genera *Lachnospiraceae* NK4A136, *Coprococcus*, and *Acetatifactor*) and increases in two Bacteroidetes OTUs (genus *Bacteroides*, and an unspecified genus within order Bacteroidales S24-7) associated with increasing Time Freezing during Acquisition (Tone 2; Figure 8B).

**Figure 9:**
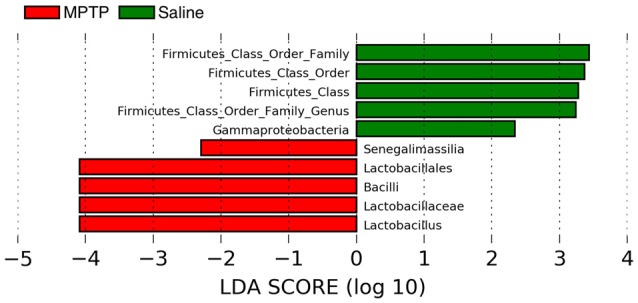
Linear discriminate analysis (LefSe) of the taxa that are significantly over-represented in the MPTP treatment (red bars) and or the control treatment (green bars).

Lastly, despite no significant difference in overall microbiome composition between MPTP and saline treatment, which could be due either to a true lack of difference or inadequate statistical power due to the number of samples analyzed, there were a handful of taxa indicative of each treatment (Figure 9) according to LefSe analysis (a hierarchical linear discriminate analysis). For both the MPTP and the saline treatments, many of the indicative taxa were within the Firmicutes phylum. Taxa within the class Bacilli (specifically the Lactobacilliales order down to the *Lactobacillus* genus) were indicative (over-represented) of the MPTP treatment, while primarily undescribed taxa that matched to the Firmicutes phylum were indicative of the saline treatment.

## Discussion

Our data show that activity in the dark phase during the first week of MPTP treatment is increased in WT and KO mice. Further, MPTP increased activity of female and male WT and KO mice in the open field. MPTP also showed genotype- and sex-dependent effects. MPTP increased the time spent exploring objects in the open field in WT, but not KO, females and males. In WT mice, MPTP improved sensorimotor function in males but impaired it in females. Further, MPTP impaired cued fear memory in WT, but not KO male mice. MPTP reduced striatal TH levels in WT and KO mice but these effects were only pronounced in males. MPTP reduced striatal β-actin levels in WT males and profoundly reduced the TH/β-actin ratio in WT females, but not males, and KO males, but not females. These findings reinforce the validity of using 2-week MPTP exposure to model motor as well as non-motor symptoms of PD. Although we decreased the dosage and exposure time of MPTP treatment from 4 weeks (Goldberg et al., 2011) to 2 weeks in the current study, we still found significant effects of MPTP treatment. Notably, KO MPTP-treated mice were protected from MPTP-induced effects in WT mice—locomotor hyperactivity, sensorimotor deficits, and impaired cue memory—highlighting a potential protective role of mGlu8 deficiency.

With regard to the altered home cage activity following MPTP treatment, the significant changes in the effects of MPTP in week 1 and week 2 indicate that continued exposure is important. The increased activity in the dark cycle of MPTP-treated mice suggests that MPTP can model circadian changes seen in PD patients. Specifically, patients show increased sleep latency, reduced sleep efficiency, and a tendency to excessive daytime sleepiness that possibly reflects pathology in the molecular clock (Breen et al., 2014). It is worthwhile to note that an alternate MPTP exposure regimen (4 mg/kg for 4 days or 22 mg/kg chronically using Alzet mini pumps) and using running/exercise wheels to assess home cage activity failed to induce any MPTP-specific alterations in circadian activity levels (Fifel et al., 2013). The different MPTP treatment regimens and the rewarding effects of wheel running (Meijer and Robbers, 2014) might have contributed to the divergent results.

WT mice treated with MPTP showed greater levels of exploratory activity in novel environments, seen in the open field and object exploration. Increased activity in MPTP-treated WT mice was also seen during the baseline of the fear conditioning training session (baseline motion). This hyperactivity may correspond to higher extracellular dopamine levels seen prior to a subsequent decline (Chesselet and Richter, 2011). The hyperactivity was associated with decreased TH levels. We have previously reported a correlation (*r* = 0.6, *p* < 0.0007) between striatal TH protein expression and DA tissue levels (Goldberg et al., 2011), suggesting in the current study that loss of TH was associated with a decrease in DA tissue levels. The same significant correlation was found between levels of DA in the striatum and loss of DA neurons in the substantia nigra. Interestingly, the hyperactivity in a novel environment seen in mice deficient in GABA_B_ receptors was associated with increased extracellular DA levels and reduced TH mRNA, but not protein, in the midbrain (Vacher et al., 2006).

WT males treated with MPTP showed improved rotarod performance. This might relate to the increased grip strength seen in MPTP-treated mice in the 4-week regimen (Sconce et al., 2015) and is reminiscent of impaired grip release seen in PD patients (Jordan et al., 1992; Gordon, 1997). Strikingly, this effect was sex-dependent; WT females treated with MPTP showed worse rotarod performance than WT females treated with saline. Importantly, mGlu8 deficiency prevented the MPTP effects in males and females, consistent with a protective effect of mGlu8 inhibition on motor function. This further supports the lack of hyperactivity in the KO mice following MPTP exposure and suggests that mGlu8 inactivation may prevent changes in locomotor performance. Interestingly, KO males treated with MPTP show both a decrease in TH levels not seen in KO females as well as a decrease in TH β-actin ratios in part due to a lack of group differences in β-actin ratios. In WT mice, the effects of MPTP on reducing TH levels were also more pronounced in males than females. However, when the ratio TH/β-actin was used as an outcome measure, the effects of MPTP were pronounced in females, but not males. Saline-treated KO females had lower TH levels and TH/β-actin ratios compared to KO males arguing that the protection seen in KO females in the rotarod performance may not be dependent on TH levels. This sex-dependent pattern of TH/β-actin ratios following MPTP in WT mice is consistent with a mouse study using 1–7 days of MPTP exposure (20 mg/kg; Ookubo et al., 2009). Interestingly, in that study, the representative western blot shown suggests decreases in both TH and β-actin levels in MPTP-treated males, similar to what we observed.

Consistent with our results, reduced TH immunoreactivity was reported in MPTP-treated WT mice (Sedelis et al., 2000). The sex-dependent effects of MPTP on β-actin levels highlight the importance of using total protein levels rather than housekeeping genes such as β-actin or α-tubulin as controls in Western blot analyses, as their levels of expression are often altered in animal models of neurodegenerative disease (for a review see Eaton et al., 2013). Our results showing effects of MPTP on β-actin levels are consistent with the reported effects of MPTP on cytoskeletal structure, including actin and α-tubulin, in cell culture (Cappelletti et al., 1991; Urani et al., 1994). Consistent with these data, gene expression of cytoskeletal proteins is also altered in the context of MPTP exposure (Cuadrado-Tejedor et al., 2005). More efforts are warranted to determine the effects of MPTP on the cytoskeleton.

MPTP-treated WT males showed impaired cued fear memory. As cued fear memory is hippocampus-independent and amygdala-dependent (Fanselow and Poulos, 2005), this might reflect MPTP-induced amygdala pathology. Hippocampus-dependent contextual fear memory was not affected in MPTP-treated male mice, consistent with another study in MPTP-treated male mice (Kinoshita et al., 2015). MPTP impairs cognitive performance on other tests as well (Yabuki et al., 2014; Zhu et al., 2015), supporting the need to consider both motor and cognitive outcome measures in developing therapeutic strategies. Along with locomotor differences, the lack of cognitive changes in the KO mice supports mGlu8 as therapeutic target in PD. In contrast, MPTP in our exposure regimen did not result in significant depressive behaviors, assessed as time spent immobile during the forced swim test (data not shown), suggesting that other PD animal models might be required to assess depressive behaviors in PD and consistent with the notion that different animal models pertinent to PD represent distinct aspects of PD (McDowell and Chesselet, 2013; Ribeiro et al., 2013; Koprich et al., 2017), Together with the sex-dependent effects of MPTP on behavioral and cognitive performance, the TH and β-actin protein levels emphasize the importance of including both sexes in the development of therapeutic targets in preclinical models of PD. This inclusion is important as women, although they have a lower risk to develop PD, might be more vulnerable to certain aspects of PD than men.

Our microbiome results indicate that there is a possible effect of MPTP treatment on the diversity of the gut microbiota. However, this significance may be largely due to a single sample with a very high diversity in the control group. Even if the difference in α-diversity is only significant due to this outlier sample, it appears that the KO mice generally have lower microbiome diversity than WT mice, implying that mGlu8 deficiency has some association, through physiology or behavior, with reduced microbiome diversity. This is unsurprising considering the widespread localization of mGlu8 in the gut (Tong and Kirchgessner, 2003; Young et al., 2008).

Furthermore, as revealed by LefSe, an increased in abundance of the genus *Lactobacillus* is indicative of MPTP treatment (see also Supplementary Figure S1). Various species within this genus have been associated with myriad effects on the nervous system, including anxiety, depression, and stress (Wang and Kasper, 2014), activation of intestinal neurons (Mao et al., 2012; Gareau, 2016), and positive and negative effects on spontaneous experimental autoimmune encephalomyelitis, an animal model of multiple sclerosis (Ezendam et al., 2008; Maassen and Claassen, 2008). Concordantly, we find that regardless of genotype or MPTP treatment, there exist significant associations with (Damier et al., 1999) microbiome α-diversity and performance on the rotarod, and (Björklund and Dunnett, 2007) microbiome composition and the amount of time frozen after the second tone in the fear learning and memory test. Together, these results indicate that specific taxa may have direct effects that promote sensorimotor learning and fear learning or that the same physiological effects that promote sensorimotor learning and fear learning also alter the assembly of the gut microbiome. Our finding that certain Bacteroidetes genera had a positive correlation with fear learning is consistent with prior work that found similar associations for cognitive performance in the water maze (Jorgensen et al., 2014). Prior work demonstrates that the microbiome interacts with the nervous system to alter behavioral performance via neural, immune, and humoral factors. Of particular relevance is the ability of glutamate to communicate between the gut and neural pathways (Mazzoli and Pessione, 2016). Based on our results, we hypothesize that MPTP’s effect on cognitive performance may be, at least in part, similarly mediated by the gut microbiome.

In summary, the genotype-specific changes in rotarod performance and cued fear memory, among others, show that KO mice do not display the same MPTP-related changes in behavior and cognitive performance as WT mice and, suggest that mGlu8 would be a worthwhile target to consider for developing PD therapeutics. However, we recognize that we cannot exclude potential contributions of developmental alterations in mGlu8 KO mice and that additional traditional pharmacological studies would be required to gain further support for validation of mGlu8 as clinical target in PD. Therefore, increased efforts are warranted to develop a specific mGlu8 antagonist, which is current not available, to determine whether acute mGlu8 inactivation can also protect against effects of MPTP when administered prior, during, or following toxin exposure. Acute mGlu8 inhibition might also be protective in other models of neurodegeneration.

## Author Contributions

JR, RD, CM and ERT designed the experiments and wrote the manuscript. ERT also analyzed the behavioral data. TA ran TH analyses and wrote the manuscript. TS and KS analyzed the microbiome data and wrote the manuscript.

## Conflict of Interest Statement

The authors declare that the research was conducted in the absence of any commercial or financial relationships that could be construed as a potential conflict of interest.

## Abbreviations

KO: knockout
mGlu: metabotropic glutamate receptor
MPTP: 1-methyl-4-phenyl-1,2,3,6-tetrahydropyridine
PD: Parkinson’s disease
TH: tyrosine hydroxylase
WT: wild-type.
