# Supplementary material for: Effects of Sub-Chronic MPTP Exposure on Behavioral and Cognitive Performance and the Microbiome of Wild-Type and mGlu8 Knockout Female and Male Mice
Source: Front Behav Neurosci. 2018 Jul 18;12:140. doi: 10.3389/fnbeh.2018.00140 (PMC6058038; doi:10.3389/fnbeh.2018.00140)

Suppl Fig 1. Increase in abundance of the genus *Lactobacillus*, indicative of MPTP as revealed by LefSe.

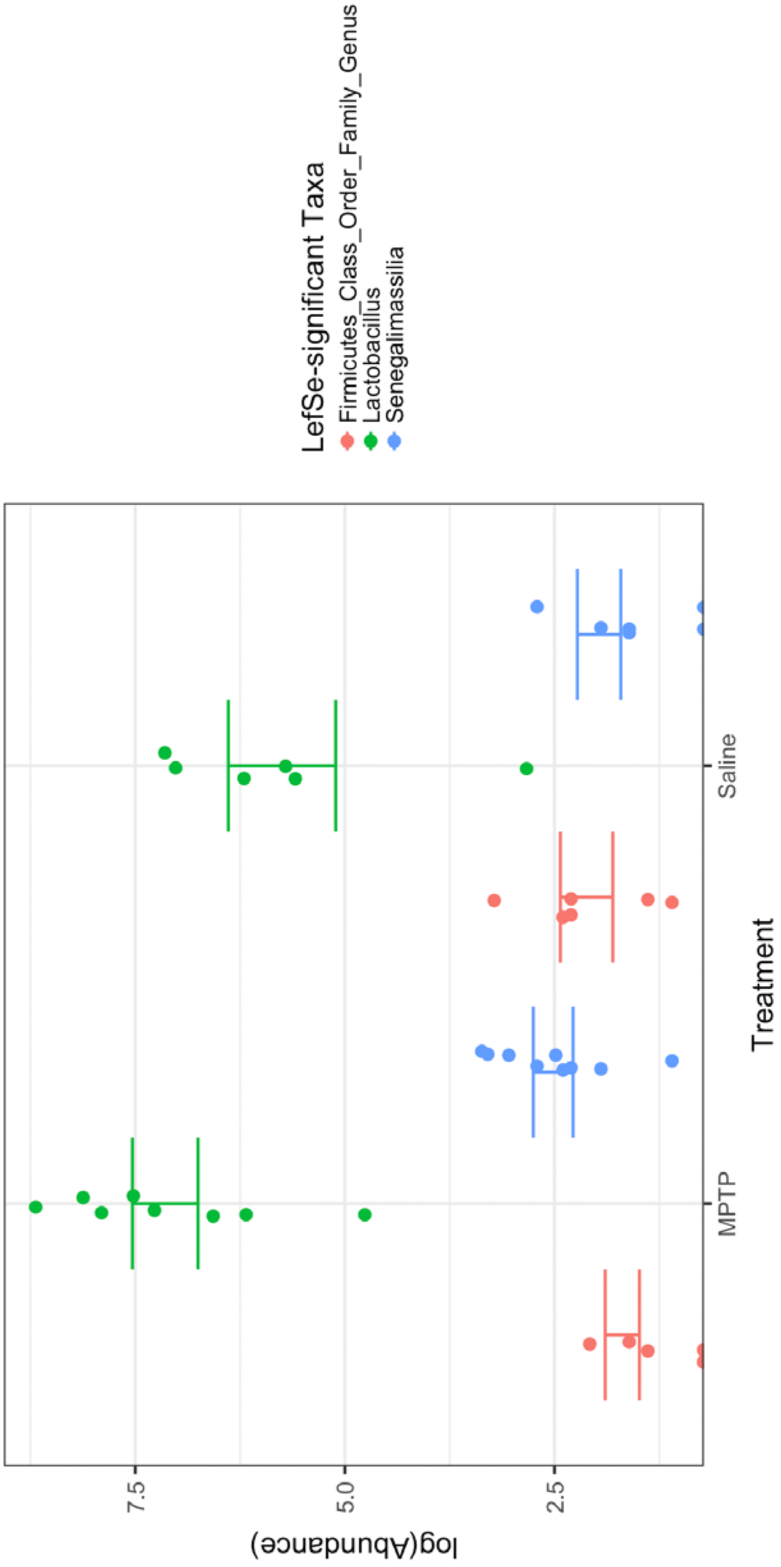

Supplement: Supplementary file 2 [file Image_1.pdf]
